# Time dependent hazard ratio estimation using instrumental variables without conditioning on an omitted covariate

**DOI:** 10.1186/s12874-021-01245-6

**Published:** 2021-03-20

**Authors:** Todd A. MacKenzie, Pablo Martinez-Camblor, A. James O’Malley

**Affiliations:** 1 Department of Biomedical Data Science, Dartmouth College, New Hampshire, USA; 2 The Dartmouth Institute for Health Policy and Clinical Practice, Dartmouth College, New Hampshire, USA

**Keywords:** Causal inference, Censoring, Semi-parametric model, Marginal model

## Abstract

**Background:**

Estimation that employs instrumental variables (IV) can reduce or eliminate bias due to confounding. In observational studies, instruments result from natural experiments such as the effect of clinician preference or geographic distance on treatment selection. In randomized studies the randomization indicator is typically a valid instrument, especially if the study is blinded, e.g. no placebo effect. Estimation via instruments is a highly developed field for linear models but the use of instruments in time-to-event analysis is far from established. Various IV-based estimators of the hazard ratio (HR) from Cox’s regression models have been proposed.

**Methods:**

We extend IV based estimation of Cox’s model beyond proportionality of hazards, and address estimation of a log-linear time dependent hazard ratio and a piecewise constant HR. We estimate the marginal time-dependent hazard ratio unlike other approaches that estimate the hazard ratio conditional on the omitted covariates. We use estimating equations motivated by Martingale representations that resemble the partial likelihood score statistic. We conducted simulations that include the use of copulas to generate potential times-to-event that have a given marginal structural time dependent hazard ratio but are dependent on omitted covariates. We compare our approach to the partial likelihood estimator, and two other IV based approaches. We apply it to estimation of the time dependent hazard ratio for two vascular interventions.

**Results:**

The method performs well in simulations of a stepwise time-dependent hazard ratio, but illustrates some bias that increases as the hazard ratio moves away from unity (the value that typically underlies the null hypothesis). It compares well to other approaches when the hazard ratio is stepwise constant. It also performs well for estimation of a log-linear hazard ratio where no other instrumental variable approaches exist.

**Conclusion:**

The estimating equations we propose for estimating a time-dependent hazard ratio using an IV perform well in simulations. We encourage the use of our procedure for time-dependent hazard ratio estimation when unmeasured confounding is a concern and a suitable instrumental variable exists.

## Background

Confounding is a threat to all observational studies. The factors that influence the outcome of interest may also influence the selection of treatment. In randomized studies, intention-to-treat estimators are generally consistent estimators of the intention-to-treat effect but are not consistent estimators of the treatment effect. Instrumental variables (IV) influence treatment choice but otherwise have no effect on the outcome (*exclusion restriction*) and are independent of any other causes of the outcome (*randomization assumption*).

Estimation of treatment effects using instrumental variables for outcomes subject to right censoring has received the attention of many applied and several theoretical studies. Stukel et al. [1] proposed an ad-hoc estimator of the hazard ratio (HR) based on a linear model. MacKenzie et al. [2] proposed a hazard ratio estimator that assumes omitted covariates have an additive effect on the hazard. Tchetgen et al. [3] proposed an estimator of an additive hazards model based on two stage residual inclusion. They also argue that if the survival curve is close to unity (e.g. above 80%) for most of the followup that their additive hazards approach can be used as a good approximation of the multiplicative hazards approach. Li et al. [4] proposed a consistent estimator of a treatment satisfying an additive hazard model. Martinussen et al. [5] derived a consistent estimator for a structural Cox model. Martínez-Camblor et al. [6] identified the role of frailties in estimation of the hazard ratio via the two-stage residual inclusion algorithm if the treatment and omitted covariate jointly satisfy a Cox model. Wang et al. [7] derived an estimator of the marginal hazard ratio using a binary instrument.

The proportionality of hazards is a key assumption in estimation of the hazard ratio. Many test statistics have been proposed to test this assumption and estimate the hazard ratio as a function of time. The most simple approach to moving beyond proportionality of hazards is a piecewise constant function. The next most simple is a linear function of time. In this paper we extend the IV based estimator of the HR we proposed in 2014 MacKenzie et al. [2] beyond proportionality of hazards. We propose a method of estimating the HR as a time-dependent function. We address two cases, (*a*) a piecewise constant HR and (*b*) a log-linear time dependent hazard ratio. We conduct Monte Carlo simulations that include the use of copulas to generate potential times-to-event. We demonstrate this method for time-dependent hazard ratio estimation to compare the effect of two vascular interventions on survival.

## Methods

### Marginal structural Cox proportional hazards model

MacKenzie et al. [2] implemented a Cox proportional hazards model for the effect of treatment on the time-to-event that is marginal with respect to any *omitted covariate*. The term marginal is used to mean that if the treatment is applied independently of the omitted covariate, the distribution of the time-to-event conditioning only on the treatment satisfies a Cox PH model, which we now elaborate on.

Let {*S*_*x*_} be the survival curve of the potential outcomes (time-to-event) if subjects receive level *x* of the exposure. In what follows we shall assume *X* is binary, e.g. 0 (no treatment) or 1 (treatment), although the methods described below are applicable to a continuously valued exposure. The structural Cox model is given by $S_{x}(t)=S_{0}(t)^{\exp \{\beta _{X}\cdot x\}}$ and it is usually written in terms of the hazard function,

$$\lambda(t;x)=\lambda_{0}(t)\cdot \exp \left\{\beta_{X}\cdot x\right\},$$

where *λ*_0_(*t*) is the *baseline hazard* function. For any covariate, *U*, which also affects the time-to-event, let *S*_*x*_(· ; *u*) be the survival curve for the potential time-to-event if a subject receives level *x* conditional on *U*=*u*. The marginal structural Cox proportional hazards model supposes that

$$\begin{array}{*{20}l} {}\mathbb E_{U}\left[S_{x}(t;\, u)\right]&= \int S_{x}(t;\, u)dF_{U}(u)=S_{x}(t)\\&\quad=S_{0}(t)^{\exp\{\beta_{X}\cdot x\}} {\text { for all }} t\geq 0\\&\quad {\text { and exposure levels }} x, \end{array} $$

where *F*_*U*_ stands for the distribution function of *U* on the studied population. The alternative to the marginal model is a conditional model that specifies a structural form for the conditional distribution given both the exposure *X* and omitted covariate *U* (e.g. multivariable Cox PH model). Due to non-collapsibility of Cox’s model [8], the marginal model that corresponds to this multivariable Cox model is not a Cox PH model.

Specifying that the marginal model is a Cox PH model has the following advantages. First, it makes no parametric assumption about how the omitted covariate *U* affects the distribution of the time-to-event: We allow for the possibility that the omitted covariate has a causal effect on the outcome without specifying a parametric model. Second, it does not require interpreting the treatment effect as conditional on a variable that is unknown (the omitted covariate). Third, this is the convention used in the reporting of randomized trials: It is unusual to analyze randomized trials using models that control for omitted covariates (such as Cox’s model with frailty), and the convention has been not to condition on measured characteristics either [9].

### Time dependent hazard ratio

Proportionality of hazards is equivalent to a hazard ratio that does not vary over time. A constant hazard ratio can be a simple and good approximation, just as a linear model may be chosen when there is evidence of some non-linearity. On the other hand there are times when the hazard ratio varies considerably and attention is focused on its time dependence. For instance, surgery may be associated with increased risks up front compared to endovascular procedures. There is a large literature on estimation of the hazard ratio as a function of time including smoothing splines [10], regression splines [11] or penalized regression splines [12] among other techniques.

### Time-to-event, exposure and instrument

Suppose the observed data consists of independent and identically distributed observations, $\left \{W_{i}, X_{i}, \Delta _{i}, T_{i}\right \}_{i=1}^{n}$ where *W*_*i*_ is the instrument (1≤*i*≤*n*) and *X*_*i*_ is the exposure level (either binary, ordinal or continuous). $\Delta _{i}=1_{T^{0}_{i}<C_{i}}$ and $T_{i}={min}(T^{0}_{i}, C_{i})$ are the event indicator and observed followup time respectively where $T^{0}_{i}$ is the focus of interest, the time-to-event, and *C*_*i*_ is the censoring time. We assume that the potential censoring times {*C*_*i*_(*x*)}_*x*=0,1_ are independent of the potential time-to-events $\left \{T^{0}_{i}(x)\right \}_{\left \{x=0,1\right \}}$.

An instrument is defined by the following properties:

1. It is associated with the exposure (*W* ⊥̸ ⊥*X*).
2. It satisfies the following two restrictions:
  - There is no effect of the instrument on the outcome except through its effect on the exposure.
  - There are no confounders of the instrument and the outcome.

The latter two statements can be summarized as the potential outcomes are independent of the instrumental variable, {*T*^0^(*x*)}_{*x*=0,1}_ ⊥ ⊥*W*.

To satisfy Assumption 1 the instrument may either be causal (i.e. it affects treatment choice) or non-causal (it is an effect of a variable that also affects exposure). Assumptions 2(a) and 2(b) are often combined by stating that conditional on *X*, the outcome and instrument are independent. The instrument, *W*, may be continuous, ordinal or binary.

For each subject there is one *potential* stochastic process {*N*_*i*_(*t*;*x*),*R*_*i*_(*t*;*x*)}_*t*≥0,*x*_ for each possible level of *x* where *R*_*i*_(*t*;*x*) is the *at risk* indicator $1_{T_{i}(x) \geq t}$, and *N*_*i*_(*t*;*x*) is the *counting process*$1_{T_{i} \leq t}$. Let *R*_*i*_(*t*)=*R*_*i*_(*t*;*X*_*i*_) and *N*_*i*_(*t*)=*N*_*i*_(*t*;*X*_*i*_).

In studies of instrumental variables another assumption that is typically introduced is homogeneity of the treatment effect and monotonicity of the effect of the instrument. In usual applications homogeneity of the treatment effect is the assumption that the effect is same for different levels of the omitted covariate, i.e. there is no interaction of the effect of the treatment and the omitted covariate. In the marginal model an estimate of the treatment effect will depend on the value of the omitted covariate. We do not specify the effect of the omitted covariate; we avoid this parameterization as the method we propose below relies on a first order linear approximation of the hazard in terms of the omitted covariate.

Another frequently addressed assumption in studies of instrumental variables is monotonicity of the effect of the instrument on the treatment. It assumes that in all subjects the instrument is positively associated with the treatment; for instance, the derivation of the complier average causal effect is dependent on it. In this study we make the monotonicity assumption, Pr [*X*≥*x*|*W*=*w*_1_]>*Pr*[*X*≥*x*|*W*=*w*_0_] if *w*_1_>*w*_0_ for all *x*.

### Estimating equations for a time dependent hazard ratio

Let *λ*_*x*_(*t*) be the hazard function corresponding to survival in the population were all subjects in that population exposed to level *x* of the exposure, i.e., *λ*_*x*_(*t*)=−*dS*_*x*_(*t*)/*S*_*x*_(*t*) which can also be written $\mathbb E[dN_{i}(t;x)|R_{i}(t;x)=1]$. We suppose that, at time *t*, the marginal (e.g. population) causal effect of having been exposed to level 1 relative to level 0 of the time-varying binary treatment *X*(*t*) is a change in the log-hazard of *β*_*X*_(*t*):

1 $$\begin{array}{@{}rcl@{}} \lambda_{1}(t) = \exp\{\beta_{X}(t)\}\cdot \lambda_{0}(t). \end{array} $$

Further, suppose that the time dependent log-hazard ratio is parameterized by *β*_*X*_(*t*;*θ*). For instance, one simple parameterization is *β*_*X*_(*t*;*θ*)=*θ*_0_+*θ*_1_·*t*.

If there is no selection bias, i.e. *X* is independent of any omitted covariate, *U*, the difference

$$N_{i}(t) - \int_{0}^{t} R_{i}(v)\cdot \exp\{\beta_{X}(v)\cdot X_{i}\} d\Lambda_{0}(v),$$

which is known as a Martingale residual (Kalb eisch and Prentice [13]), has an expected value of zero and is independent of *X*$\left (\Lambda _{0}(v)=\int _{0}^{v} \lambda _{0}(s)ds\right)$. This implies that

$$\begin{array}{*{20}l}{}\mathbb E&\left[X_{i}\cdot\left(N_{i}(t) - \int_{0}^{t} R_{i}(v)\cdot \exp\{\beta_{X}(v;\theta)\cdot X_{i}\}d\Lambda_{0}(v)\right)\right]\\&\quad=0\quad \forall t>0.\end{array} $$

Moreover,

$$\begin{aligned} \mathbb E\left[ \int_{0}^{t}\phi(v)\cdot X_{i}\cdot\left(dN_{i}(v) - R_{i}(v)\cdot \exp\left\{\beta_{X}(v;\theta)\cdot X_{i}\right\}d\Lambda_{0}(v)\right) \right]=0 \end{aligned} $$

for any real function *ϕ*(·).

This equation suggests that an estimator of *β*_*X*_(*t*;*θ*) is that ${\hat \beta _{X}(t;\theta)}$ for which

2 $$ \begin{aligned} 0=\sum_{i=1}^{n} \int_{0}^{\tau} \phi_{k}(v)\cdot X_{i}\cdot \left[ dN_{i}(v) - R_{i}(v)\cdot \exp\left\{{\hat \beta_{X}(v;\theta)}\cdot X_{i}\right\} d\Lambda_{0}(v)\right]  \end{aligned}  $$

where *τ* is a suitably large point of time (e.g. max1≤*i*≤*n*{*t*_*i*_}), *K* is the dimension of the parameter *θ*=(*θ*_1_,⋯,*θ*_*K*_) and $\left \{\phi _{k}(t)\right \}_{k=1}^{K}$ are suitably selected real functions. These functions should be chosen to minimize the variance of the resulting estimator which motivates the choices *ϕ*_*k*_(*t*)=*∂β*_*X*_(*t*;*θ*)/*∂θ*_*k*_.

As *Λ*_0_(*t*) is unknown we propose to solve for it using the estimating equations

3 $$\begin{array}{@{}rcl@{}} 0\,=\,\sum_{i=1}^{n} \left[N_{i}(t) \,-\, \int_{0}^{t} R_{i}(v)\cdot \exp\left\{{\hat \beta_{X}(v;\theta)}\cdot X_{i}\right\}d{\hat \Lambda_{0}}(v) \right] \end{array} $$

for all *t* which is solved by the Breslow estimator (Lin [14])

4 $$\begin{array}{@{}rcl@{}} d \hat \Lambda_{0}(v)=\frac{\sum_{i=1}^{n} dN_{i}(v)}{\sum_{i=1}^{n} R_{i}(v)\cdot \exp\left\{{\hat \beta_{X}(v;\theta)}\cdot X_{i}\right\}}. \end{array} $$

Substitution of (4) into (2) yields the score equations for the partial likelihood (Cox [15]) for a time-dependent hazard ratio (Abrahamowicz et al. [11]). That is, the estimating equations (2) are equivalent to the method of maximum partial likelihood estimation of a time-dependent hazard ratio. If *X* is endogenous (confounded), then *X*_*i*_ is not necessarily independent of the Martingale residual

$$N_{i}(t) - \int_{0}^{t} R_{i}(v)\cdot \exp\left\{\beta_{X}(v;\theta)\cdot X_{i}\right\}d\Lambda_{0}(v)$$

and therefore the estimating Eq. (2) are biased.

If *W* is an instrumental variable then it is approximately independent of the counting process conditional on *X*, or equivalently, the IV is independent of the Martingale residual. A heuristic justification starts by writing the causal hazard function conditional on an omitted covariate *U* that affects both the treatment and the time-to-event. We approximate the causal hazard function by a linear Taylor expansion, E [*dN*_*i*_(*t*)|*R*_*i*_(*t*)=1]= exp{*β*_*X*_(*v*;*θ*)·*X*_*i*_}*dΛ*_0_(*v*)+*ϕ*(*t*)[*U*−*μ*_*U*_(*t*)] where *μ*_*U*_(*t*) is the expected value of the omitted covariate among people at risk at time *t*. Then equating to zero the correlation of the instrument with the counting process leads to the following estimating equation *θ*,

5 $$ \begin{aligned} 0=\sum_{i=1}^{n} \int_{0}^{\tau} W_{i} \cdot \frac{\partial}{\partial \theta_{k}} \beta_{X}(v;\theta)\cdot \left [dN_{i}(v) - R_{i}(v)\cdot \exp\left\{{\hat \beta_{X}(v;\theta)}\cdot X_{i}\right\} d\Lambda_{0}(v)\right ] . \end{aligned}  $$

If we substitute the Breslow estimator for *Λ*_0_(*v*) into the latter equation it results in the estimating equation

6 $$ \begin{aligned} 0=\sum\limits_{i=1}^{n}\int_{0}^{\tau} \phi_{k}(v)\cdot \left[ W_{i}-\frac{\sum\limits_{j=1}^{n} W_{j}\cdot R_{j}(v)\cdot \exp \left\{{\beta_{X}(v;\theta) }\cdot X_{j}\right\}}{\sum\limits_{j=1}^{n}R_{j}(v)\cdot \exp \left\{{\beta_{X}(v;\theta) }\cdot X_{j}\right\}}\right] dN_{i}(v).  \end{aligned}  $$

If the time-dependent function is constant, i.e. *β*_*X*_(*v*;*θ*) equals a scalar *θ* this is the same estimating equation proposed in MacKenzie et al. [2].

### IV estimation for piecewise proportional hazards

In applications, categorization of follow-up time is favored as an approach to assessing time-dependence of the hazard ratio because of its simplicity. For instance, one can make statements such as, in the first month the hazard ratio was 1.5 but after one month was 0.5. The time-frame is categorized into intervals $\left \{\tau _{i-1},\tau _{i}\right \}_{i=1}^{K}$ for 0=*τ*_0_<*τ*_1_<…<*τ*_*K*_=*τ* and within each window the hazard ratio is assumed to be constant, *β*_*X*_(*t*;*θ*)=*θ*_*i*_ for *τ*_*i*−1_≤*t*<*τ*_*i*_. The partial likelihood estimator of a step function time dependent hazard ratio is equivalent to applying the partial likelihood estimator of the proportional hazards model within each time window. For instance, to estimate the hazard ratio in the interval [*τ*_*i*−1_,*τ*_*i*_) exclude all subjects eliminated from risk before time *τ*_*i*−1_ and censor any events after time *τ*_*i*_ (1≤*i*≤*K*). For this piecewise constant hazard ratio the IV based estimator proposed in (6) is equivalent to applying the IV based method of MacKenzie et al. [2]. The R function supplied in that paper can be used accordingly to implement this approach for each time window (https://github.com/toddamackenzie/Instrumental-Variable-Hazard-Ratio-Estimation).

### IV estimation for linear time dependent log hazard ratio

Any parameterization of the hazard ratio could be implemented with the instrumental variable estimating equations we propose. We chose to illustrate the approach using a log-linear time-dependent model of the hazard ratio, *β*_*X*_(*t*;*θ*)=*θ*_0_+*θ*_1_·*t*. In this parameterization exp{*θ*_0_} is the hazard ratio at inception and exp{*θ*_1_} is the multiplicative change in the hazard ratio per unit time. The two parameters *θ*_0_ and *θ*_1_ can be estimated using the equations

7 $$ \begin{aligned} 0=\sum\limits_{i=1}^{n}\int_{0}^{\tau} \left[ W_{i}-\frac{\sum\limits_{j=1}^{n} W_{j}\cdot R_{j}(v)\cdot \exp\left\{{\beta_{X}(v;\theta)}\cdot X_{j}\right\}}{\sum\limits_{j=1}^{n}R_{j}(t)\cdot \exp\left\{{\beta_{X}(v;\theta)}\cdot X_{j}\right\}}\right] dN_{i}(v),  \end{aligned}  $$

and

8 $$ \begin{aligned} 0=\sum\limits_{i=1}^{n}\int_{0}^{\tau} v\cdot\left[ W_{i}-\frac{\sum\limits_{j=1}^{n} W_{j}\cdot R_{j}(v)\cdot \exp \left\{{\beta_{X}(v;\theta) }\cdot X_{j}\right\}}{\sum\limits_{j=1}^{n}R_{j}(v)\cdot\exp\left\{{\beta_{X}(v;\theta) }\cdot X_{j}\right\}}\right] dN_{i}(v),  \end{aligned}  $$

respectively.

### Monte Carlo simulations

We evaluated the behavior of the estimating equations we propose in (6) under two scenarios for the marginal time-dependent hazard ratio; *i*) a three piece constant hazard ratio, and *ii*) a log linear time-dependent hazard ratio. In the first scenario, the estimation method is equivalent to applying the approach of MacKenzie et al. [2] in early, middle and late follow-up settings, and that is how we implement and report the results. These simulations are novel in that the 2014 paper did not consider estimation of the hazard ratio later in follow-up, such as where the survival curve dips below 75%. Because a much greater proportion of the study will have either experienced the outcome event or been censored by that time, it is reasonable to expect that the performance of the method could differ over this time-period compared to the early follow-up period. For the log-linear function of time we consider a range of intercepts and a range of slopes. Specific details follow.

#### Comparators

In addition to evaluating the bias of our estimator for the stepwise constant and log-linear hazard ratio models, we evaluate the performance of the partial likelihood estimator. For the stepwise hazard model, because it is a proportional hazards model in each of the three periods, we also evaluate the performance of two other estimators designed for proportional hazards. First, we have evaluated the approach of Wang et al.[7] which estimates the hazard ratio for a marginal structural model like ours. Because Wang et al’s approach is designed for a binary instrument, we dichotomize the continuous instrument of our simulations at the median. Second, we have evaluated the performance of the estimator of Martínez-Camblor et al. [6] which estimates the hazard ratios of a Cox model for the multivariable effect of the treatment and the omitted covariate; that is, it conditions on the omitted covariate.

#### Simulation methods

We have conducted extensive simulations to evaluate the bias of the estimator we propose. Our simulation uses a copula to generate times-to-event that depend on an omitted covariate and treatment in such a way that the marginal distribution of the potential outcomes, treated and untreated, satisfy a model with the specified time-dependent hazard ratio. The settings and parameter values for the simulation are listed in Tables 1 and 2.

**Table 1:** Simulation Variables: *Φ*(·) is the cumulative distribution functions for the standard normal distribution and *F*_1_ is the cumulative distribution function for the time-to-event that has log-hazard of *β*_1_,*β*_2_,*β*_3_ in periods 1,2 and 3 respectively

Variable	Symbol	Generation
Correlated Uniform Marginals	(*Y*_0_,*Y*_1_)	∼ copula(*ρ*)
Omitted Covariate	U	*Φ*^−1^(*Y*_0_)
Instrumental Variable	W	∼ N(0,1)
Potential Outcome if not Exposed	*T*_0_	−log(*Y*_0_)
Potential Outcome if Exposed	*T*_1_	$F_{1}^{-1}(Y_{1})$
Exposure	X	Pr [*X*=1]= logistic (*α*_*UX*_*U*+*α*_*WX*_*W*)

**Table 2:** Parameters are constant within a dataset but vary between datasets

Element	Value Set
log Odds Ratio U vs X: *α*_*UX*_	(−log(10), log(10))
log Odds Ratio W vs X: *α*_*WX*_	(log(2), log(50)
*ρ*: correlation of copula	0.50, 0.99
Copula family	Gaussian, Clayton, Gumbel
Stepwise log Hazard Ratio Period 1 *β*_1_	[−ln(3),−ln(3)+log(6)/24,…, ln(3)]
Stepwise log Hazard Ratio Period 2 *β*_2_	[−ln(3),−ln(3)+log(6)/24,…, ln(3)]
log-linear HR intercept	$[ -1, -0.8,\dots,1]$
log-linear HR slope over time	$[ -0.5, -0.4,\dots, 0.5]$
Sample size of each dataset	1000
Censoring frequency	0.50
Number of events	500

Before each dataset is constructed the parameters above are randomly drawn with equal probability from the value set

The steps of the simulation are:

1. Randomly generate the bivariate random variable, (*Y*_0_,*Y*_1_), from a bivariate copula., i.e. *Y*_0_ and *Y*_1_ are uniformly distributed but not independent.
2. Let the omitted covariate be the standard normal deviate obtained as *U*=*Φ*^−1^(*Y*_0_).
3. Randomly generate a continuous instrument, *W*, from the standard normal distribution.
4. Randomly generate a binary exposure indicator *X* using a logistic link with intercept of zero (yields equal proportions of treated and untreated) and log odds ratios, *α*_*W*_ and *α*_*U*_, for the association of *W* and *U* respectively.
5. Obtain potential time-to-events *T*(0)=− log(*Y*_0_) (unit exponential distribution) and $T(1)=F_{1}^{-1}(Y_{1})$ where *F*_1_ is the cumulative distribution function whose hazard equals the specified time-dependent hazard ratio
6. Right censor the times-to-event using a uniform distribution whose scale is set to achieve the specified censoring rate.

In our Monte Carlo simulation the confounding (endogeneity) is created by (i) setting the omitted covariate as a monotonic function of the potential outcome under no treatment, which is correlated with the potential outcome under treatment (as controlled by the bivariate copula using either Pearson or Kendal correlations of 0.50 or 0.99) and (ii) randomly generating the treatment indicator based on the omitted covariate (as well as the instrument). Therefore, the level of confounding by the omitted covariate is controlled by a single parameter, the odds ratio, exp (*α*_*UX*_) relating *U* (the untreated time-to-event transformed to have a standard normal distribution) to *X*. For instance, if *α*_*UX*_=0 there is no confounding. Before the generation of each dataset it was randomly drawn from a Uniform distribution on the interval log(1/10), log(10). Values above log(5) were considered positive confounding, and values below − log(5) were considered negative confounding.

The stepwise constant time-dependent hazard ratio took values as specifed in Table 2. For each simulated dataset we randomly sampled with replacement from these grids for the early, mid and late follow-up hazard ratios. The early corresponded to approximately that time period up to which survival curve was above 90%, the middle 90% down to 75%, and late survival of 75% or less. The log-linear time-dependent hazard ratio was generated based on the randomly drawn intercept and slope as specified in Table 2.

Complete R code is available at: https://github.com/toddamackenzie/Instrumental-Variable-Hazard-Ratio-Estimation, for users interested in conducting the same simulations.

### Simulation results for early, mid and late estimators of the HR

**Fig. 1:**
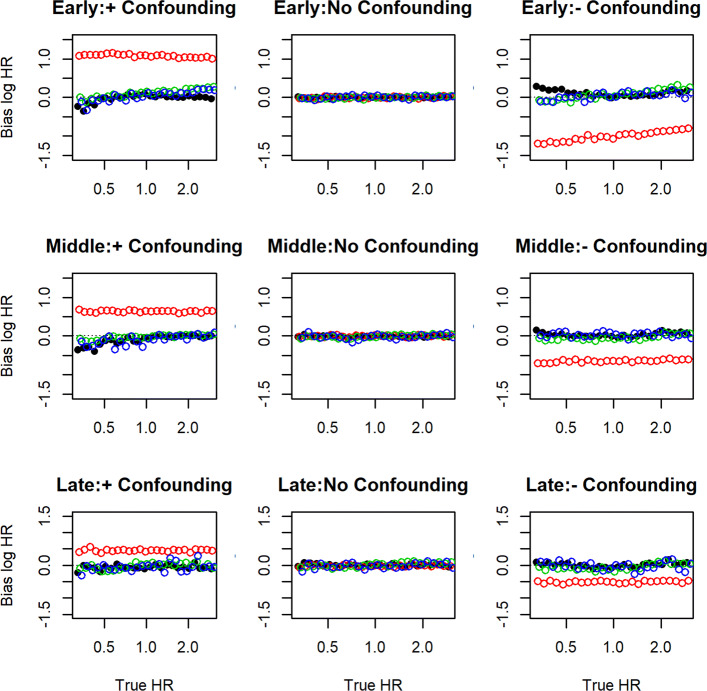
Simulation Results for estimating three-piece time-dependent hazard ratio when the instrument is strong: The nine panes represent each combination of early, mid and late follow-up, with (*i*) strong positive confounding, (*ii*) no confounding and (*iii*) strong negative confounding. Each pane overlays scatterplots of the median estimated bias versus the true hazard ratio, our IV based estimator in black, the standard Cox MPLE in red, the estimator of Wang in green and the estimator of Martinez-Camblor et al. in blue. Each point represents the median of over 1000 estimates obtained from that many randomly generated datasets

Figure 1 shows the results of the simulations when the instrument is strong, which we defined as an odds ratio between the treatment and the instrument that exceeds 5. Each red point represents the median of over 1000 maximum partial likelihood estimates of the stepwise hazard ratio. The partial likelihood estimators are clearly subject to bias from confounding, which decreases as follow-up increases. Each black point represents the median of over 1000 estimates using the instrument and estimating equations we propose. The green and blue points represent the median of the distribution of the estimators of Wang et al. [7] and Martínez-Camblor et al. [6]. The instrumental variable based estimator we propose is unbiased as an estimator of the marginal hazard ratio in each of the three periods, with a slight tendency toward bias by confounding for large and small hazard ratios; that is, the magnitude of the bias increases as the HR moves away from null and in the direction opposite of the confounding. The estimators of Wang et al. [7] and Martínez-Camblor et al. [6] demonstrate similarity and slightly better accuracy.

**Fig. 2:**
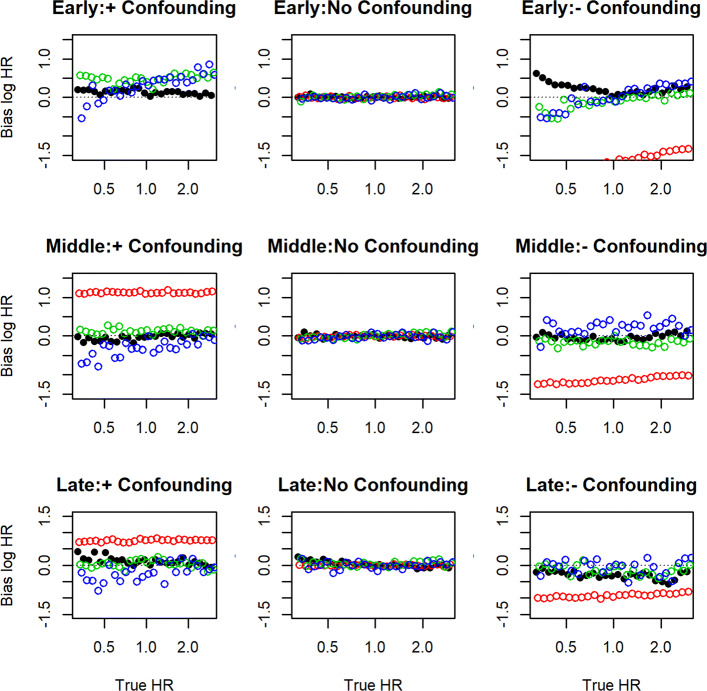
Simulation Results for estimating three-piece time-dependent hazard ratio when instrument is weak: See Fig. 1 caption for details

The bias was affected by the strength of the instrument; for each doubling of the odds ratio between the treatment and the instrument, the bias reduced by approximately 5%. Figure 2 shows the results restricted to instruments for which the odds ratio between treatment and instrument was less than 5. The results are similar to those reported for the scenario of a strong instrument. The bias of our estimator did not vary with respect to choice of copula, or the correlation between the treated and untreated potential outcomes (results not reported).

### Simulation results for log-Linear time dependent HR

**Fig. 3:**
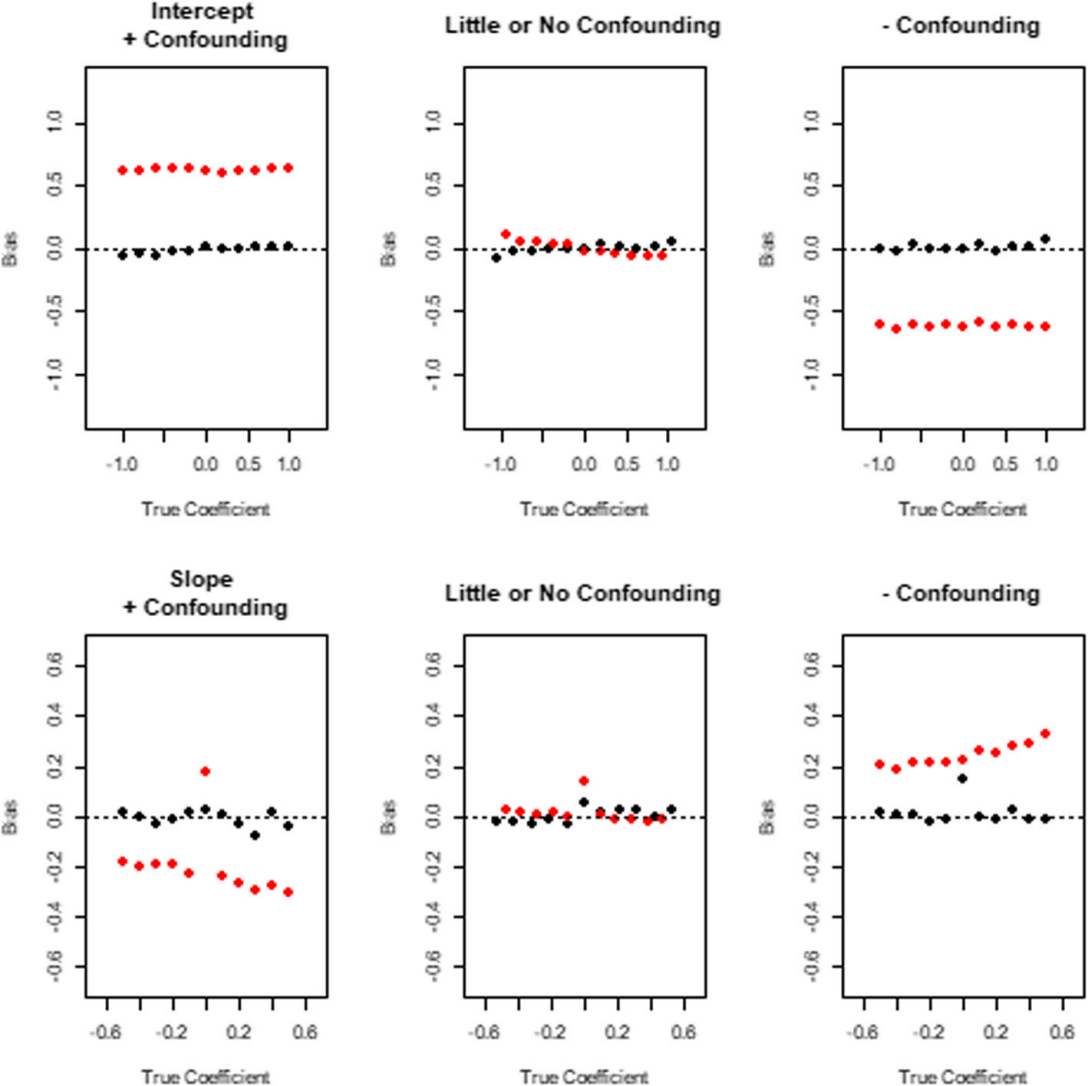
Simulation Results for Estimating a Log-Linear Time Dependent Hazard Ratio: The top 3 panes represent estimation of the intercept parameter of the log-linear model for (*i*) strong positive confounding, (*ii*) no confounding and (*iii*) strong negative confounding, while the bottom three panes represent results for estimation of the slope parameter. Each panel overlays scatterplots of the median bias (estimated hazard ratio minus true coefficient) versus the true coefficient, our IV based estimator in black, and the Cox MPLE in red. Each point represents the median of over 1000 estimates (from that many simulated datasets)

Figure 3 shows the results of the simulations for estimation of the log-linear hazard ratio. The top row demonstrates that the intercept term of the log-linear hazard ratio is estimated with very little bias using the instrumental variable estimating equation we propose (blue points represent median of over 1000 estimates from that many simulated datasets), unlike the maximum partial likelihood estimators (points in red). Results from the estimation of slope parameter of the log-linear hazard ratio are found in the bottom row, which again show that the IV based estimating equation we propose yields unbiased estimators (blue points), unlike the partial likelihood estimator (in red) with the exception that the IV based estimator of the slope is biased when the true slope is zero. The estimators of Wang et al. [7] and Martínez-Camblor et al.[6] are not designed for log-linear estimators or any time-dependent hazard ratio beyond piecewise constant.

The simulation findings did not change with respect to the choice of the copula, the correlation between the treated and untreated potential outcomes, or the strength of the instrument.

## Real-world application

**Fig. 4:**
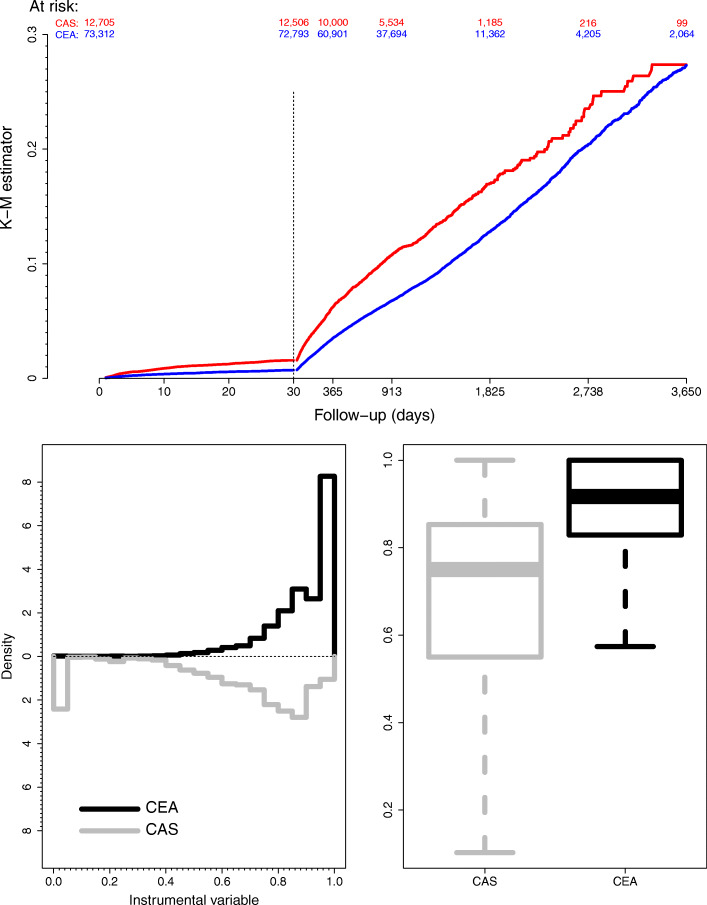
Kaplan-Meier estimates for the survival curves (top). Dashed vertical line stands for the perioperative period (30 days) while numbers indicate the patients at risk each 2 years within the CAS and CEA groups). At botton, histograms for the instrumental variable distributions (left) and box-plot (right) for both the CEA and the CAS groups. Notice that a value of zero in the IV implies that in the hospital this patient received the surgery, no CEA surgeries were performed in the 12 months prior to that patient. On the other hand, a value of 1 implies that, in this hospital, in the last 12 months, all patients received CEA

We illustrate our method to address a comparative effectiveness question in patients with carotid stenosis. We use data from the nationwide Vascular Quality Initiative, VQI, (http://www.vascularqualityinitiative.org) to compare the composite outcome of death or stroke following intervention between those undergoing endarterectomy (CEA) and those undergoing carotid stenting (CAS). The data consists of 73,312 patients who received CEA and 12705 who received CA during the years 2003-2016. The number of events was 8,005 of which 730 occurred in the first 30 days, 2498 in months 1 through 12 and 4,777 after the first year. This example has been previously considered by Columbo et al. [16]. Figure 4 (top) shows the Kaplan-Meier estimates for the survival curves on both the CEA and the CAS groups.

The population of patients who undergo CEA may not be the same as those who undergo CAS, and therefore any estimator of comparative efficacy may be biased by confounding. Therefore we utilize an instrumental variable approach. The instrument we employ is the center level relative frequency of CEA versus CAS procedures over the 12 months prior to the current patient. A value of zero indicates that the patient received the surgery in a facility which during the prior 12 months, has not performed CEA. The validity of this instrument is argued in Martínez-Camblor et al. [17]. Figure 4, bottom, depicts the instrument’s distribution (histograms at left and box-plot at right) in both groups.

**Fig. 5:**
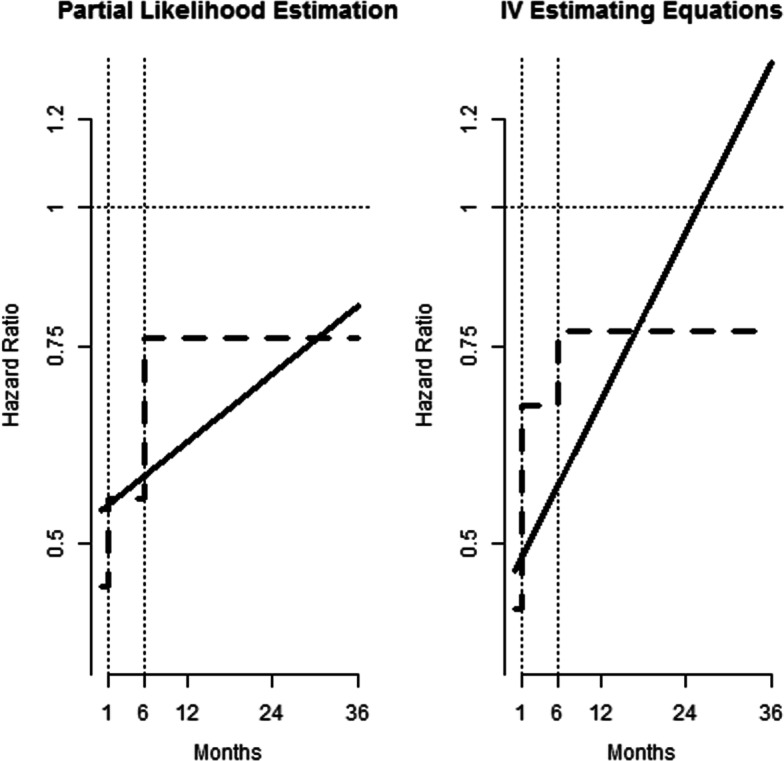
Hazard Ratios Comparing Risk of Stroke or Death Between CSA and CEA: The left pane shows partial likelihood estimators of a 3 piece time-dependent constant hazard ratio (steps at 1 and 6 months, in a dashed line) and a log-linear time-dependent hazard ratio (solid line). The pane on the right shows the corresponding estimators based on the instrumental variable we proposed

Figure 5 shows estimates of the hazard ratio as a function of time. The left panel contains the partial likelihood estimators of the stepwise constant hazard ratio and the log-linear hazard ratio. After consulting with physicians and a visual inspection of the Kaplan-Meier survival curve, we chose the cut-offs of 1 month (30 days) and 6 months. In the first month the partial likelihood estimate of the hazard ratio comparing CEA to CAS is 0.46 (95%CI: 0.39 to 0.54), while it is 0.55 (95% CI: 0.48 to 0.62) in months 2 through 6, and considerably closer to unity thereafter at 0.75 (95%CI: 0.71 to 0.82). The corresponding estimates based on our instrumental variable approach are similar in the first interval, 0.44 (95%CI: 0.31 to 0.61), although the confidence interval is wider as expected for IV based estimators. The IV based estimator of the hazard ratio in the middle interval is closer to unity, 0.66 (95%CI: 0.50 to 0.88). The estimator in the final interval is 0.77 (95%CI: 0.65 to 0.91).

The model for the hazard ratio as a log-linear function of time indicates the same pattern of the hazard ratio moving toward unity as time progresses. Both the partial likelihood estimator and the IV based estimator indicate the hazard ratio begins at approximately 0.5 but the IV based estimator reaches unity earlier. The observed results at both early and late follow-up were quite close in the standard and proposed methodologies suggesting that, in average, the potential covariates affecting the survival have a minor impact on the effect of the treatment. Between the first and six months, the difference is around 20% indicating that in this period some omitted covariates spuriously enhance the observed effect of the treatment.

## Discussion

We have proposed a method for using instruments in the estimation of time-dependent hazard ratios. The framework is general enough to accommodate any parameterization for a time-dependent hazard ratio. Like maximum partial likelihood estimation of Cox’s proportional hazards model it does not require a parameterization of the baseline hazard. We have illustrated our approach using two forms for a time-dependent hazard ratio, (*i*) a piecewise constant hazard ratio and (*ii*) a log linear function of time.

Our approach focuses on time-dependent hazard ratios that are marginal with respect to the omitted covariate. That is, just like estimators of treatment effects in randomized studies, the model we employ does not explicitly condition on the omitted covariate although it is motivated by a linear approximation of a hybrid hazard function in which the dependence on the omitted covariate is additive and the dependence on treatment and the observed covariates is multiplicative. Our approach is analogous to estimators of population averaged parameters such as generalized estimating equations. We encourage analysts to estimate both marginal models like ours and models that condition on the omitted covariate. A disadvantage of the conditional model is that it conditions on intangible characteristics, that is unmeasured characteristics, that somehow affected the treatment selection.

We conducted extensive simulations to evaluate our estimator of the piecewise hazard ratio and the log-linear hazard ratio. For the former, which reduces to a proportional hazards model for any of the time windows (in which the hazard ratio is constant) we compared our approach to the partial likelihood estimator, the approach of Martínez-Camblor et al. [6] and the approach of Wang et al. [7]. For the log-linear hazard ratio model, we compared our approach to the partial likelihood estimator. The simulations indicated our estimator of a piecewise hazard ratio has some bias toward the null which is comparable to the bias of Martínez-Camblor et al. [6] and Wang et al. [7]. Our simulations indicate our estimator of the log-linear hazard ratio has little bias, especially in comparison to maximum partial likelihood estimation when confounding by omitted covariates is present.

While we do not address it, this method generalizes readily to multivariable models without much additional complexity. That is, measured covariates can be incorporated into the model.

The method we propose has some notable limitations. We do not believe it is a consistent estimator because we derived our estimating equations by substituting for the baseline hazard the Breslow estimator without justification of why this is the ideal estimator using an instrumental variable. In addition, it is based on an approximation of the hazard as an additive function of the omitted covariate.

We believe our estimator can be improved by utilizing inverse probablity weighting in our estimating equations. In particular, like the approach of Wang et al. [7] and Huling et al. [18] one should weight the subjects at risk at any time *t* using the instrumental variable to make those subjects at risk generalizable to the entire population, i.e. those at risk at time *t*=0, at which time the instrument is orthogonal to the omitted covariates.

## Conclusion

The estimating equations we propose for estimating a time-dependent hazard ratio using an IV perform well in simulations. We encourage the use of our procedure for time-dependent hazard ratio estimation when unmeasured confounding is a concern and a suitable instrumental variable exists.

## Data Availability

The code for this method and the simulations is available at: https://github.com/toddamackenzie/Instrumental-Variable-Hazard-Ratio-Estimation. The data used in the example was accessed from Centers for Medicare and Medicad Servies under contract and is not available for sharing.

## Abbreviations

IV: Instrumental Variable
HR: Hazard Ratio
